# Patients' Willingness of First Visit in Primary Medical Institutions and Policy Implications: A National Cross-Sectional Survey in China

**DOI:** 10.3389/fpubh.2022.842950

**Published:** 2022-04-01

**Authors:** Jin Li, Ning Zhao, Haiyan Zhang, Hui Yang, Jia Yang

**Affiliations:** ^1^School of Public Health, Capital Medical University, Beijing, China; ^2^Department of Health Education, Beijing Huairou Hospital of University of Chinese Academy of Sciences, Beijing, China; ^3^Beijing Key Laboratory for Pediatric Diseases of Otolaryngology Head and Neck Surgery, Beijing Children's Hospital, Capital Medical University, National Center for Children's Health, Beijing, China

**Keywords:** primary medical institutions, first visit, hierarchical medical system, patient willingness, China

## Abstract

**Background:**

The Chinese hierarchical treatment system expects patients to first visit primary medical institutions (PMIs), and patients' willingness determined their utilization of primary health care. The aim of this study was to explore the factors associated with patients' willingness to make their first visit to PMIs.

**Methods:**

We employed multistage stratified sampling and convenience sampling to administer questionnaires to 1,507 patients in Beijing, Qinghai, and Fujian. Patients' willingness of first visit in PMIs was analyzed using Chi-square test and binary logistic regression.

**Results:**

Of the 1,507 participants in the survey, 55.1% were willing to make their first visit in PMIs. Fewer patients in Beijing (17.6%) are willing to make their first visit in PMIs than those in Qinghai (71.9%) and Fujian provinces (72.0%). Binary logistic regression analysis revealed that higher recognition of the community first visit policy and higher satisfaction with the medical technology of PMIs are associated with patients' willingness of first visit in PMIs.

**Conclusions:**

Due to differences in local economic conditions, medical resources, and policy formulation, there are differences among provinces in patients' willingness of first visit in PMIs. To increase patients' rate of visits in PMIs, it is important to improve service capacity and quality of PMIs and change residents' attitudes for PMIs.

## Introduction

The World Health Organization issued the Alma-Ata Declaration in 1978. The document identifies primary healthcare as the core of integrated health services and regards primary healthcare as the basis for a sustainable development of healthcare systems (1). There is presently a three-tier health care system in China (primary, secondary, and tertiary medical facilities), and different levels of facilities have different functions and positions (2). Medical institutions of higher levels have more medical resources and can provide more comprehensive health services (3). Primary medical institutions (PMIs), including community health service centers /stations, township health centers and village clinics, mainly provide basic public health services and primary care, such as disease prevention, chronic disease management, health education and the treatment of common and frequent diseases (4). By the end of 2020, there are 35,365 community health service centers/stations, 35,762 township health centers, and 608,828 village health clinics in China (5). Due to lack of trust of patients for PMIs and the demand for quality healthcare services, the majority of patients prefer to bypass primary care and go to high-level hospitals (6–8).

It is very common for patients to overutilize high-level hospitals healthcare services but underutilize primary healthcare services in China (9). In 2017, outpatient visits of tertiary hospitals accounted for 45.17% of the total number of outpatient visits, compared to 15.93% for community health centers (10). Over 70% of patients with general and chronic diseases choose to consult doctors at tertiary hospitals (11). To improve the utilization efficiency of medical resources in PMIs and relieve the pressure of high-level hospitals, the Chinese government has formulated a series of policies such as hierarchical medical system and medical insurance differentiated reimbursement policy (12, 13). The purpose is to guide residents to make their first visit in PMIs. Hierarchical medical system aims to require patients to choose PMIs as their first site of treatment, then transfer to high-level hospitals for further treatment based on their condition. Meanwhile, high-level hospitals transfer inpatients in stable conditions to PMIs for rehabilitation (14). But this policy did not strictly constrain community-based first visit, and residents are still free to choose their ideal health care facility for consultation. Medical insurance differential reimbursement policy guides patients to seek medical treatment in an orderly manner by appropriately raising the proportion of medical insurance reimbursement in PMIs. The Chinese government expects to adjust the layout of the medical service system and rationalize the allocation and utilization of medical resources through relevant policies to finally solve the problem of “difficult and expensive medical treatment.” But the community first visit system is not really working and exists in name only (15). Medical resources are more abundant in urban areas, and thus regardless of the severity of the disease, urban patients prefer to access secondary and tertiary hospitals instead of PMIs (16, 17). According to data from the *2020 China Statistical Yearbook of Health*, the number of consultations in PMIs accounted for 61.87% of total consultations in 2010, but decreased to 53.17% in 2020 (18).

Patients' willingness determines their utilization of primary health care. Previous researches focused on the people's preference for primary health care and influencing factors. For example, models of service, medical costs, satisfaction, family income, characteristics of provider and medical experience would influence patients' choice of medical institutions (19–23). First, most studies are limited by geographic areas, and the scope of the studies is in one province or one city, lacking cross-provincial studies (3, 20, 22–24). Second, few studies have studied whether residents' knowledge of the policy influences their willingness or choice to seek treatment (25, 26). Cross-provincial studies are useful for the formulation of relevant medical policies. It is essential for policymakers to understand the impact of policies on patients' choice of medical institutions. However, there has not been a study on patients' willingness of first visit in PMIs, and the impact of policy awareness on first visits. Therefore, this study selected the three provinces based on regional distribution and economic development level for analysis. The study has two objectives: (1) to investigate patients' willingness of first visit in PMIs; and (2) to explore the variables that are associated with patients' willingness of first visit in PMIs, especially the relevant policy variables. Based on the results of this study, we discuss how to improve patients' willingness of first visit in PMIs, in order to provide reference for the government to develop a hierarchical medical system.

## Materials and Methods

### Study Design and Data Sources

**Figure 1:**
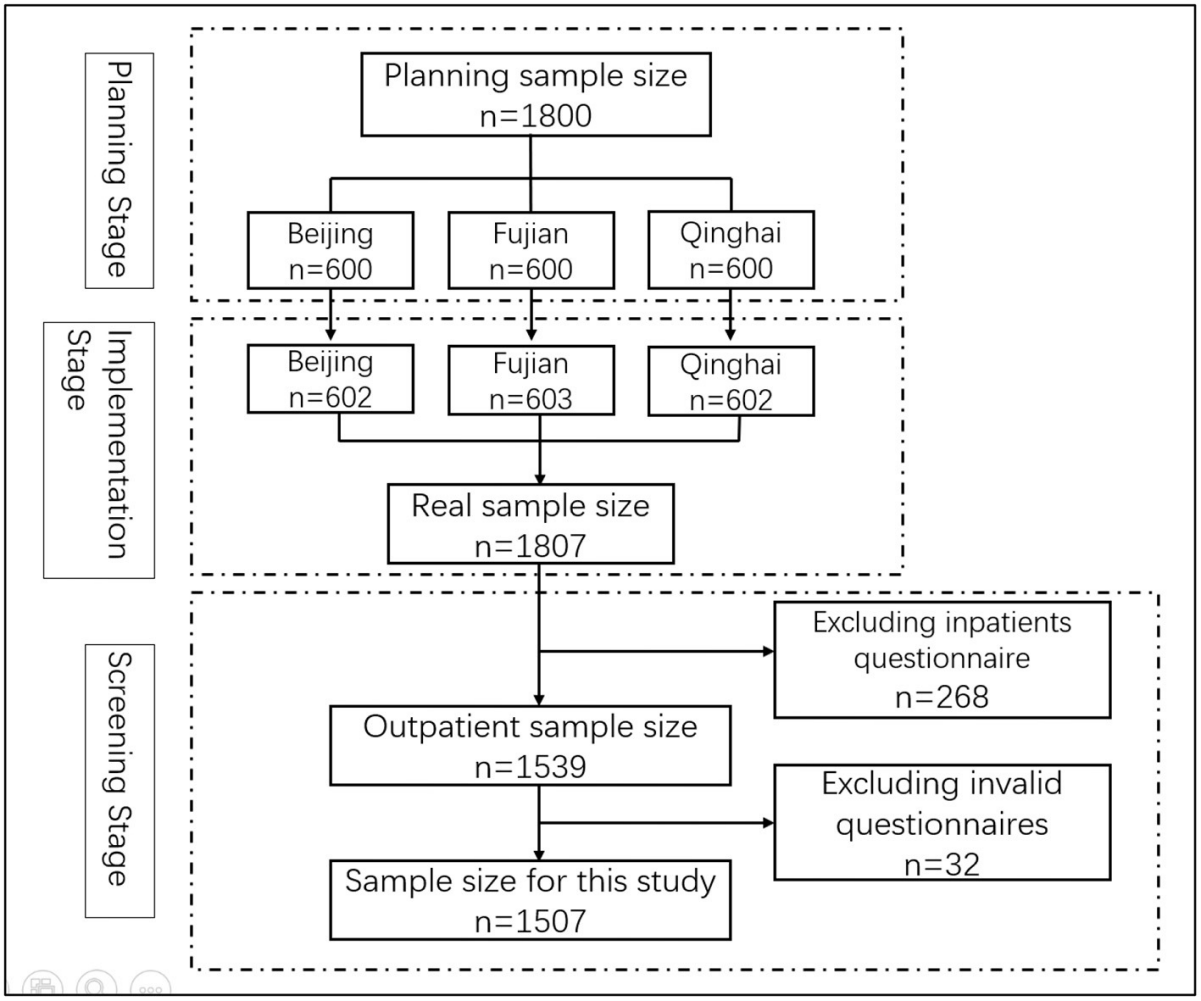
Flow chart of the selection of respondents.

The study is a cross-sectional survey, and multi-stage stratified sampling and convenience sampling were used to choose research subjects. Firstly, according to the geographical location and economic development level, three provinces were chosen (Beijing, Fujian and Qinghai provinces). Secondly, according to the progress of the implementation of the hierarchical medical system and economic development level, two cities were selected from each province, and a total of six cities were selected. The formula for calculating the sample size for each city patient is $n=\frac{Z^{2}P\left(1-P\right)}{E^{2}}$, *Z* = 1.96. E is the error value, *E* = 5%; *P* is the ratio of visits at community health centers to total visits in 2020, *P* = 26%; *n* = 296. Therefore, we planned to select 300 patients in each city. Third, with the support of the Chinese Hospital Association, each city selected two tertiary hospitals, two secondary hospitals, and two community healthcare centers. The sample size of tertiary hospitals, secondary hospitals and community health centers in each city was 100. Fourth, convenience sampling was used to survey patients. 50 patients in each medical institution were selected for the investigation. In total, 1,807 questionnaires were distributed. Based on previous experience, there are very few inpatients in PMIs, so only outpatients were analyzed in this study. After excluding inpatients and invalid questionnaires s, a total of 1,507 outpatients were included in the research sample, and the valid response rate was 83.40%. The sample size selection process was showed in Figure 1.

The questionnaire was self-designed on the basis of literature research and expert consultation. After designing the questionnaire, we looked for respondents to conduct a pre-investigation to test the reliability and validity of the questionnaire and revised it based on the survey results. After several revisions, the contents and structure of the questionnaire were determined. Finally, the questionnaire was converted into an electronic questionnaire to survey. Data were collected via face-to-face interviews with trained interviewers, and respondents were given a survey incentive to ensure the response rate and validity of the data. Meanwhile, to ensure the quality of the data, only patients over 18 years old were included. Before handing out the questionnaire, we stated the study purpose and content to all respondents, obtained their informed consent, and committed that their privacy was protected. This study was conducted from March to May 2019. According to the study design, only parts of the questions in the questionnaire were included as variables.

### Dependent Variable

In this study, the dependent variable is the patients' willingness of first visit in PMIs (In this questionnaire, community health service centers/stations refer to PMIs). It was measured by a question: “are you willing to make your first visit in the community and then be referred to a higher-level hospital via the community health center/station?” The response was divided into 5 options: a. very unwilling; b. more unwilling; c. indifferent; d. more willing; e. very willing. We converted the five-dimensional ordered variables to binary variables based on published studies (12, 27–29). Options a, b, and c were combined into “unwilling”; options d and e were combined into “willing.”

### Independent Variables

The independent variables were divided into three parts. Part 1 is the sociodemographic characteristics, including province, gender, age, education, registered permanent residence, chronic disease status, length of residence in the city, household annual income and average monthly medical expense. Part 2 is the attitude toward PMIs, including experience of PMIs visits in the past year, degree of satisfaction with the medical technology of PMIs and degree of satisfaction with the service attitude of PMIs. Part 3 is the level of understanding of relevant policies, including understanding of the first visit policy, recognition of the community first visit policy, understanding of the medical insurance differential reimbursement policy, and influence of the medical insurance differential reimbursement policy (see Supplementary File 1).

### Statistical Analysis

The software SPSS 26.0 was used to analyze data. Descriptive statistics (frequency and percentage) were used to describe the sociodemographic characteristics of patients, and the Chi-square test was used to analyze patients' willingness of first visit in PMIs. Those statistically significant variables in the Chi-square test were included as independent variables in the logistic regression model, and the factors associated with first visit willingness were further analyzed. The differences were regarded as to be statistically significant when *P*-value <0.05.

## Results

### Sociodemographic Characteristics and Willingness of First Visit in PMIs of All the Participants

After excluding outliers, a total of 1,507 patients were included in this study (83.40% valid rate). Table 1 presents the sociodemographic distribution characteristics of the patients. Of the 1,507 patients, 523 (34.7%) patients were interviewed in Qinghai, among whom 54.7% were females. The majority (55.4%) of patients were under 45 years old. Almost half (49.5%) of the patient's household registration were out-of-town. The great majority of patients are well-educated: 31.8% of patients have a high school degree and 62.2% have a college degree or above. Most patients (72.0%) lived in the city for more than 2 years. The largest proportion of respondents have a household annual income of <80,000 yuan, and 64.0% of respondents have average monthly medical expenditures between 301 and 800 yuan. For chronic disease status, 96.7% of respondents have chronic diseases.

**Table 1:** Patients' sociodemographic characteristics and univariate analysis of patients' willingness of first visit in PMIs.

**Variables**	**The willingness of first visit to PMIs** ***N*** **(%)**			
	**Unwilling *N* = 677 (44.9)**	**Willing *N* = 830 (55.1)**	**Total *N* = 1,507, *N* (%)**	**χ^2^**	***P***
**Part 1 Sociodemographic**					
**Province**				384.967	**<0.001**
Beijing	385 (82.4)	82 (17.6)	467 (31.0)		
Fujian	145 (28.0)	372 (72.0)	517 (34.3)		
Qinghai	147 (28.1)	376 (71.9)	523 (34.7)		
**Gender**				2.491	0.115
Male	322 (47.1)	361 (52.9)	683 (45.3)		
Female	355 (43.1)	469 (56.9)	824 (54.7)		
**Age (years)**				3.816	0.282
<45	392 (46.9)	443 (53.1)	835 (55.4)		
45–54	158 (41.5)	223 (58.5)	381 (25.3)		
55–64	87 (42.4)	118 (57.6)	205 (13.6)		
≥65	40 (46.5)	46 (53.5)	86 (5.7)		
**Education**				0.473	0.789
Junior or below	41 (45.6)	49 (54.4)	90 (6.0)		
Senior high school	209 (43.6)	270 (56.4)	479 (31.8)		
Bachelor or above	427 (45.5)	511 (54.5)	938 (62.2)		
**Registered permanent residence**				5.688	0.058
The city's downtown	190 (43.4)	248 (56.6)	438 (29.1)		
The city's suburbs	164 (50.8)	159 (49.2)	323 (21.4)		
Out-of-town	323 (43.3)	423 (56.7)	746 (49.5)		
**Length of residence in the city (years)**				3.331	0.189
<1	66	61	127 (8.4)		
1–2	125	170	295 (19.6)		
≥2	486	599	1,085 (72.0)		
**Household annual income (yuan)**				6.301	**0.043**
<80,000	298 (41.7)	417 (58.3)	715 (47.4)		
80,000–150,000	334 (48.3)	357 (51.7)	691 (45.9)		
≥150,000	45 (44.6)	56 (55.4)	66 (4.4)		
**Average monthly medical expense (yuan)**				11.461	**0.003**
≤ 300	100 (35.8)	179 (64.2)	279 (18.5)		
301–800	455 (47.2)	510 (52.8)	965 (64.0)		
>800	122 (46.4)	141 (53.6)	263 (17.5)		
**Chronic disease status**				15.324	**<0.001**
Yes	641 (44.0)	816 (56.0)	1,457 (96.7)		
No	36 (72.0)	14 (28.0)	50 (3.3)		
**Part 2 Attitude toward PMIs**					
**Degree of satisfaction with the medical technology of PMIs**				69.461	**<0.001**
Not satisfied	20 (35.7)	36 (64.3)	56 (3.7)		
Less satisfied	268 (36.5)	466 (63.5)	734 (48.7)		
Generally	257 (61.2)	163 (38.8)	420 (27.9)		
More satisfied	84 (47.5)	93 (52.5)	177 (11.7)		
Very satisfied	48 (40.0)	72 (60.0)	120 (8.0)		
**Degree of satisfaction with the service attitude of PMIs**				24.083	**<0.001**
Not satisfied	32 (39.5)	49 (60.5)	81 (5.4)		
Less satisfied	114 (40.3)	169 (59.7)	283 (18.8)		
Generally	220 (40.4)	325 (59.6)	545 (36.2)		
More satisfied	240 (54.4)	201 (45.6)	441 (29.3)		
Very satisfied	71 (45.2)	86 (54.8)	157 (10.4)		
**Experience of PMIs visits in the past year**				1.245	0.264
Yes	565 (44.3)	710 (55.7)	1,275 (84.6)		
No	112 (48.3)	120 (51.7)	232 (15.4)		
**Part 3 Level of understanding of relevant policies**					
**Understanding of the community first visit policy**				0.497	0.481
Yes	71 (47.7)	78 (52.3)	149 (9.9)		
No	606 (44.6)	752 (55.4)	1,358 (90.1)		
**Recognition of the community first visit policy**				110.397	**<0.001**
Not recognize	224 (34.3)	429 (65.7)	653 (43.3)		
Mildly recognize	135 (40.8)	196 (59.2)	331 (22.0)		
Moderate	213 (61.4)	134 (38.6)	347 (23.0)		
Partly recognize	89 (71.8)	35 (28.2)	124 (8.2)		
Completely recognize	16 (30.8)	36 (69.2)	52 (3.5)		
**Understanding of the medical insurance differential reimbursement policy**				15.088	**<0.001**
Yes	131 (36.1)	232 (63.9)	363 (24.1)		
No	526 (47.7)	598 (52.3)	1,144 (75.9)		
**Influence of the medical insurance differential reimbursement policy**				52.499	**<0.001**
No impact	10 (20.8)	38 (79.2)	48 (3.2)		
Less impact	115 (37.0)	196 (63.0)	311 (20.6)		
Moderate	217 (39.8)	328 (60.2)	545 (36.2)		
More impact	235 (55.7)	187 (44.3)	422 (28.0)		
Greatest impact	100 (55.2)	81 (44.8)	181 (12.0)		

*The bold P values indicates that the difference is statistically significant*.

This study found that 830 (55.1%) of the 1,507 respondents were willing to first visit in PMIs and be referred to secondary or tertiary hospital through PMIs if necessary. 90.1% of respondents were understanding of the first visit policy and 43.3% of respondents said they cannot recognize it. 24.1% of patients were aware of the medical insurance differential reimbursement policy. In the past year, 84.6% of patients had experience in PMIs. 19.7% of patients were satisfied with the technology of PMIs and 39.7% of patients were satisfied with the service attitude (more satisfied and very satisfied were defined as satisfied).

### Sociodemographic Characteristics, Attitude, and Understanding of Relevant Policies and Their Relationships With Patients' Willingness of First Visit in PMIs

The Chi-square test was applied to check the relationship between all variables and willingness of first visit in PMIs. The results showed that there were statistically significant differences in the willingness of patients to first visit PMIs for province (*P* < 0.001), household annual income (*P* = 0.043), chronic disease status (*P* < 0.001), average monthly medical expense (*P* = 0.003), recognition of the community first visit policy (*P* < 0.001), understanding of the medical insurance differential reimbursement policy (*P* < 0.001), influence of the medical insurance differential reimbursement policy (*P* < 0.001), degree of satisfaction with the medical technology of PMIs (*P* < 0.001), and degree of satisfaction with the service attitude of PMIs (*P* < 0.001).

### Predictors of Patients' Willingness of First Visit in PMIs

Firstly, binary logistic regression model was tested for goodness-of-fit. The results of Hosmer–Lemeshow tests showed *P* > 0.10; binary logistic regression model was considered to be good fitting. The results of binary logistic regression analysis are presented in Table 2. Compared with patients in Qinghai, patients in Beijing were more unwilling to first visit in PMIs (OR = 0.097, *P* < 0.001). Recognition of the community first visit policy is associated with patients' willingness of first visit in PMIs. Compared with patients who completely recognize the community first visit policy, patients who partly (OR = 0.292, *P* = 0.004), moderately (OR = 0.334, *P* = 0.004), mildly (OR = 0.407, *P* = 0.019), and not recognize (OR = 0.421, *P* = 0.021) the policy were more unwilling to first visit PMIs. It means that other groups of patients had a lower willingness to first visit PMIs compared to those who strongly recognized of the community first visit policy. Compared with patients who were very satisfied with the medical technology of the PMIs, patients with general satisfaction were more unwilling to first visit PMIs (OR =0 .593, *P* = 0.034).

**Table 2:** Binary logistic regression analysis of patients' willingness of first visit in PMIs.

**Variables**	**β**	***P***	**OR**	**95%CI**	
				**Lower limit**	**Upper limit**
**Province (Ref: Qinghai)**
Beijing	−2.334	**<0.001**	0.097	0.067	0.140
Fujian	−0.024	0.880	0.977	0.718	1.328
**Household annual income (yuan) (Ref:** **>150,000)**
<80,000	−0.324	0.220	0.723	0.431	1.214
80,000–150,000	−0.223	0.401	0.800	0.475	1.347
**Chronic disease status (Ref: No)**
Yes	−0.217	0.569	0.805	0.381	1.700
**Average monthly medical expense (yuan) (Ref:** **>800)**
≤ 300	0.386	0.076	1.471	0.960	2.253
301–800	−0.100	0.555	0.905	0.648	1.262
**Recognition of the community first visit policy (Ref: completely recognize)**
Not recognize	−0.865	**0.021**	0.421	0.202	0.879
Mildly recognize	−0.899	**0.019**	0.407	0.191	0.865
Moderate	−1.096	**0.004**	0.334	0.158	0.709
Partly recognize	−1.229	**0.004**	0.292	0.127	0.674
**Understanding of the medical insurance differential reimbursement policy (Ref: No)**
Yes	0.119	0.449	1.127	0.827	1.534
**Influence of the medical insurance differential reimbursement policy (Ref: Greatest impact)**
No impact	0.541	0.195	1.718	0.757	3.901
Less impact	−0.177	0.450	0.838	0.529	1.326
Moderate	0.108	0.615	1.115	0.730	1.701
More impact	−0.060	0.784	0.941	0.611	1.450
**Degree of satisfaction with the medical technology of PMIs (Ref: Very satisfied)**
Not satisfied	−0.160	0.674	0.852	0.405	1.794
Less satisfied	−0.063	0.788	0.939	0.595	1.483
Generally	−0.523	**0.034**	0.593	0.365	0.962
More satisfied	−0.065	0.817	0.937	0.539	1.628
**Degree of satisfaction with the service attitude of PMIs (Ref: Very satisfied)**
Not satisfied	0.073	0.818	1.075	0.580	1.995
Less satisfied	0.185	0.429	1.203	0.760	1.904
Generally	0.196	0.353	1.217	0.804	1.841
More satisfied	0.194	0.384	1.214	0.785	1.877

*The bold P values indicates that the difference is statistically significant*.

### Patients' Sociodemographic Characteristics, Attitude, and Understanding of Relevant Policies and Their Relationships With Different Provinces

To further explore the disparities in other aspects among patients from different provinces, we conducted the univariate analysis (Table 3). The results showed that there were statistical significance among patients from different provinces for age (*P* = 0.008), education (*P* = 0.011), registered permanent residence (*P* = 0.016), household annual income (*P* < 0.001), chronic disease status (*P* < 0.001), degree of satisfaction with the medical technology of PMIs (*P* < 0.001), degree of satisfaction with the service attitude of PMIs (*P* < 0.001), understanding of the community first visit policy (*P* = 0.004), recognition of the community first visit policy (*P* < 0.001), understanding of the medical insurance differential reimbursement policy (*P* < 0.001), and influence of the medical insurance differential reimbursement policy (*P* < 0.001).

**Table 3:** Univariate analysis of patients' sociodemographic, attitudes and understanding of relevant policies in different provinces.

**Variables**	**Provinces** ***N*** **(%)**			
	**Beijing *N* = 467 (31.0)**	**Fujian *N* = 517 (34.3)**	**Qinghai *N* = 523 (34.7)**	**Total *N* = 1,507, *N* (%)**	**χ^2^**	***P***
**Gender**					0.630	0.730
Male	218 (31.9)	234 (34.3)	231 (33.8)	683 (45.3)		
Female	249 (30.2)	283 (34.3)	292 (35.2)	824 (54.7)		
**Age (years)**					17.340	**0.008**
<45	276 (33.1)	263 (31.5)	296 (35.4)	835 (55.4)		
45–54	113 (29.7)	144 (37.8)	124 (32.5)	381 (25.3)		
55–64	44 (21.5)	82 (40.0)	79 (38.5)	205 (13.6)		
≥65	34 (39.5)	28 (32.6)	24 (27.9)	86 (5.7)		
**Education**					12.981	**0.011**
Junior or below	20 (22.2)	25 (27.8)	45 (50.0)	90 (6.0)		
Senior high school	137 (28.6)	172 (35.9)	170 (35.5)	479 (31.8)		
Bachelor or above	310 (33.0)	320 (43.1)	308 (32.8)	938 (62.2)		
**Registered permanent residence**					12.234	**0.016**
The city's downtown	148 (33.8)	146 (33.3)	144 (32.9)	438 (29.1)		
The city's suburbs	116 (35.9)	111 (34.4)	96 (29.7)	323 (21.4)		
Out-of-town	203 (27.2)	260 (34.9)	283 (37.9)	746 (49.5)		
**Length of residence in the city (years)**					5.630	0.229
<1	41 (32.3)	44 (34.6)	42 (33.1)	127 (8.4)		
1–2	75 (25.4)	106 (35.9)	114 (38.6)	295 (19.6)		
≥2	351 (32.4)	367 (33.8)	367 (33.8)	1,085 (72.0)		
**Household annual income (yuan)**					44.866	**<0.001**
<80,000	164 (22.9)	262 (36.6)	289 (40.4)	715 (47.4)		
80,000–150,000	264 (38.2)	218 (31.5)	209 (30.2)	691 (45.9)		
≥150,000	39 (38.6)	37 (36.6)	25 (24.8)	101 (6.7)		
**Average monthly medical expense (yuan)**					9.085	0.059
≤ 300	72 (25.8)	102 (36.6)	105 (37.6)	279 (18.5)		
301–800	304 (31.5)	341 (35.3)	320 (33.2)	965 (64.0)		
>800	91 (34.6)	74 (28.1)	98 (37.3)	263 (17.5)		
**Chronic disease status**					58.202	**<0.001**
Yes	427 (29.3)	513 (35.2)	517 (35.5)	1,457 (96.7)		
No	40 (80.0)	4 (8.0)	6 (12.0)	50 (3.3)		
**Degree of satisfaction with the medical technology of PMIs**						144.844	**<0.001**
Not satisfied	9 (16.1)	14 (25.0)	33 (58.9)	56 (3.7)		
Less satisfied	144 (19.6)	315 (42.9)	275 (37.5)	734 (48.7)		
Generally	209 (49.8)	105 (25.0)	106 (25.2)	420 (27.9)		
More satisfied	72 (40.7)	41 (23.2)	64 (36.2)	177 (11.7)		
Very satisfied	33 (27.5)	42 (35.0)	45 (37.5)	120 (8.0)		
**Degree of satisfaction with the service attitude of PMIs**					121.854	**<0.001**
Not satisfied	12 (14.8)	39 (48.1)	30 (37.0)	81 (5.4)		
Less satisfied	64 (22.6)	104 (36.7)	115 (40.6)	283 (18.8)		
Generally	128 (23.5)	201 (36.9)	216 (39.6)	545 (36.2)		
More satisfied	222 (50.3)	125 (28.3)	94 (21.3)	441 (29.3)		
Very satisfied	41 (26.1)	48 (30.6)	68 (43.3)	157 (10.4)		
**Experience of PMIs visits in the past year**					3.538	0.171
Yes	400 (31.4)	445 (34.9)	430 (33.7)	1,275 (84.6)		
No	67 (28.9)	72 (31.0)	93 (40.1)	232 (15.4)		
**Understanding of the community first visit policy**					10.844	**0.004**
Yes	58 (38.9)	57 (38.3)	34 (22.8)	149 (9.9)		
No	409 (30.1)	460 (33.9)	489 (36.0)	1,358 (90.1)		
**Recognition of the community first visit policy**					309.511	**<0.001**
Not recognize	84 (12.9)	274 (42.0)	295 (45.2)	653 (43.3)		
Mildly recognize	81 (24.5)	130 (39.3)	120 (36.3)	331 (22.0)		
Moderate	194 (55.9)	70 (20.2)	83 (23.9)	347 (23.0)		
Partly recognize	89 (71.8)	24 (19.4)	11 (8.9)	124 (8.2)		
Completely recognize	19 (36.5)	19 (36.5)	14 (26.9)	52 (3.5)		
**Understanding of the medical insurance differential reimbursement policy**					263.684	**<0.001**
Yes	62 (17.1)	252 (69.4)	49 (13.5)	363 (24.1)		
No	405 (35.4)	265 (23.2)	474 (41.4)	1,144 (75.9)		
**Influence of the medical insurance differential reimbursement policy**					237.757	**<0.001**
No impact	2 (4.2)	19 (39.6)	27 (56.3)	48 (3.2)		
Less impact	32 (10.3)	183 (58.8)	96 (30.9)	311 (20.6)		
Moderate	134 (24.6)	192 (35.2)	219 (40.2)	545 (36.2)		
More impact	205 (48.6)	80 (19.0)	137 (32.5)	422 (28.0)		
Greatest impact	94 (51.9)	43 (23.8)	44 (24.3)	181 (12.0)		

*The bold P values indicates that the difference is statistically significant*.

**Figure 2:**
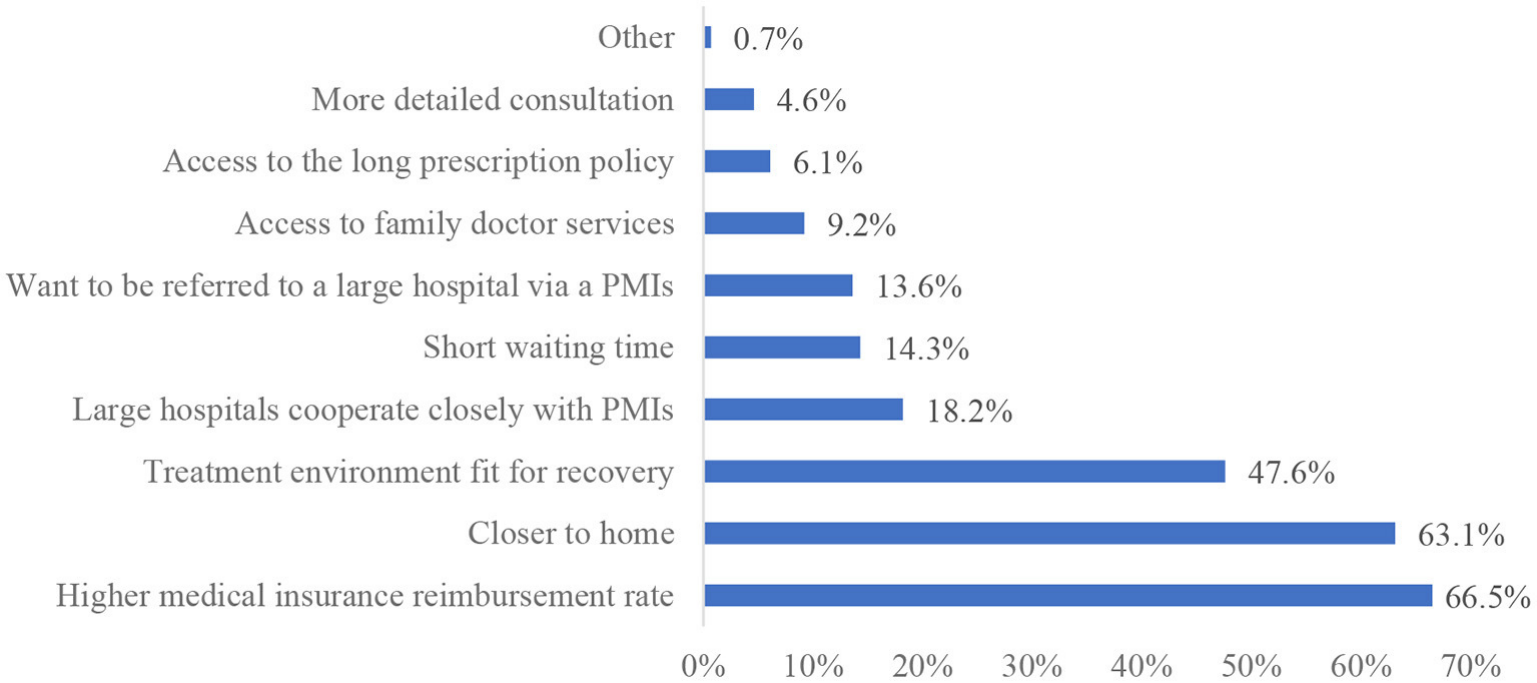
Reasons for patients' willingness for first visit in PMIs.

**Figure 3:**
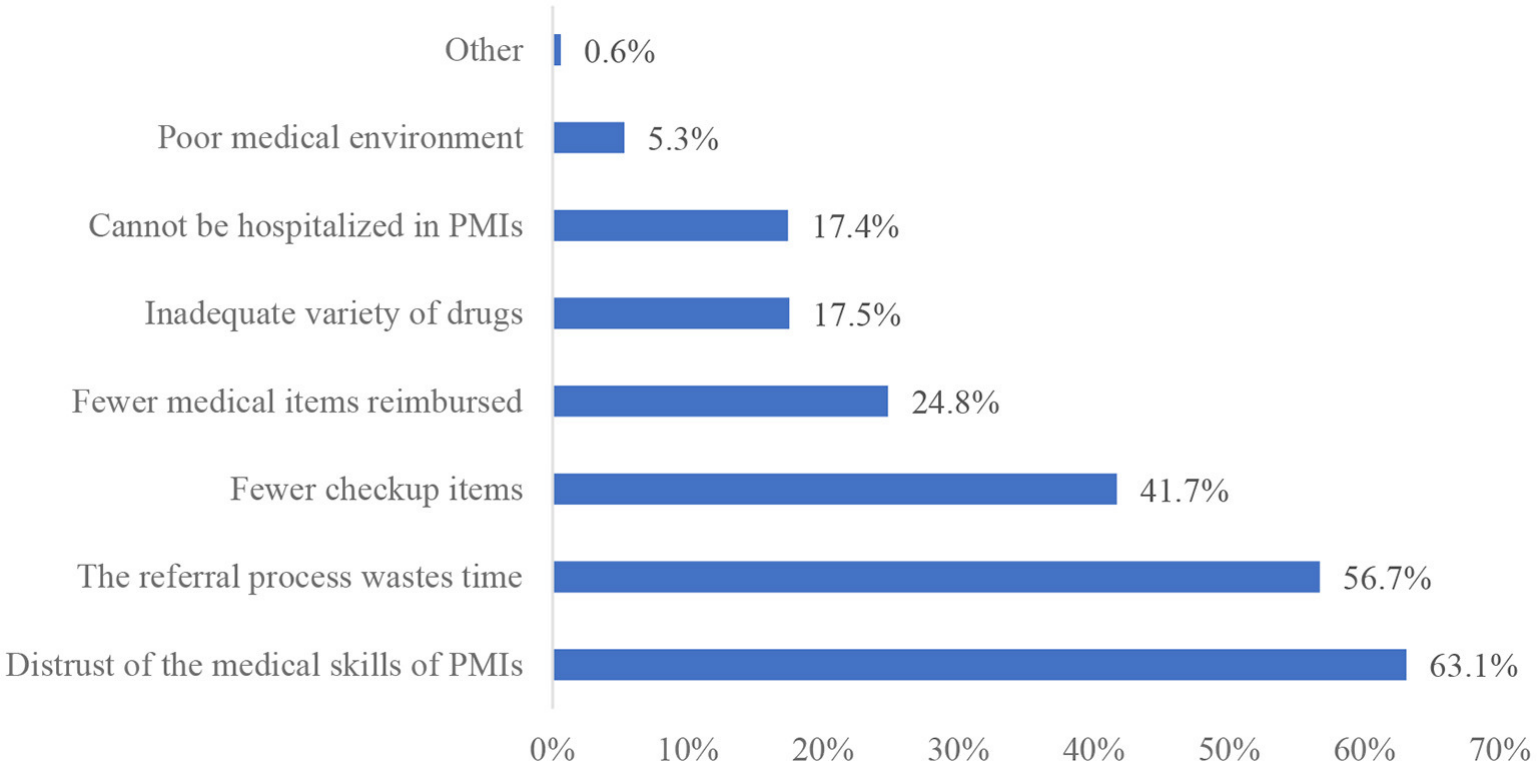
Reasons for patients' unwillingness for first visit in PMIs.

Figures 2, 3 shows the reasons why patients were willing/unwilling to make their first visit in PMIs. Higher medical insurance reimbursement rate (66.5%), closer to home (63.1%), and treatment environment fit for recovery (47.6%) are top 3 reasons for patients' willingness to go to PMIs. Distrust of the medical skills of PMIs (63.1%), the referral process wastes time (56.7%), and fewer checkup items (41.7%) are top 3 reasons for patients' unwillingness to go to PMIs.

## Discussion

To the best of our knowledge, this is the first study examining the willingness of outpatients in multiple provinces to make their first visit to PMIs. We found that patients with high recognition of the first visit policy and high satisfaction with the medical technology of PMIs were more willing to make their first visit in PMIs, and that patients in Beijing were more unwilling to make their first visit in PMIs. In this study, 55.1% of the patients were willing to make their first visit in PMIs and be referred to higher level hospitals if necessary. This result has not reached the expected goal of policy formulated by Chinese government in 2015 (≥70%) (30), and is similar to the results of a study conducted in Hubei in 2021 (55.22%) (31), and higher than that of a study conducted in nine tertiary hospitals in Shanghai in 2019 (48.4%) (32). Fewer patients are willing to first visit PMIs, which leads to low utilization of medical resources in PMIs and overutilization of medical resources in high level hospitals. There are several studies from other countries that have reached similar conclusions (33–36). A study from the United States showed that rural patients also bypass rural hospitals and choose large urban hospitals for consultations (33). A study from India found that most patients (especially hypertensive patients) bypassed primary health care and chose private medical facilities for usual care because of the low quality of primary health care (35). Our analysis shows the major reasons for patients' willingness of first visit in PMIs are higher reimbursement rate of health insurance and closer distance to home. This is consistent with the results of previous studies (20, 31, 37). For example, the policy of Qinghai Province stipulated that patients who are referred to secondary or higher hospitals through PMIs can enjoy higher reimbursement rate of medical insurance.

The results showed that patients in Qinghai Province were more willing to choose PMIs for their first visit than patients in Beijing. 17.6% of patients in Beijing are willing to choose PMIs for consultation, as compared to 71.9% in Qinghai Province. This finding is significantly lower than that of the 2019 study in Beijing by Song et al. (17.6 vs. 60.44%) (38). After preliminary analysis, we suggest that the large difference in the proportion of patients' willingness of first visit in PMIs is probably due to the different sources of research subjects in the two studies. All the samples of Song et al. were from community health service centers/stations, while those of this article were from tertiary, secondary hospitals and community health service centers. Many studies have shown that medical resource availability, transportation convenience, medical care quality, and socioeconomic factors significantly influence on patients' choice of medical institutions (39–41). Beijing is a developed economic city with a lot of high-quality medical resources, while Qinghai Province is an undeveloped economic city with fewer medical resources. The 2020 data have shown that the Gross Domestic Product Per Capita (GDPPC) in Beijing was 164,889 yuan, while Qinghai Province was 50,819 yuan (42). There were 106 tertiary hospitals and 158 secondary hospitals in Beijing, while only 24 tertiary hospitals and 88 secondary hospitals in Qinghai Province (18). Hence, patients' willingness of first visit in PMIs is influenced by economic development and medical resources in different provinces. In some economically developed provinces in eastern China, the inequality in medical resource allocation between urban hospitals and PMIs has led to an “inverted triangle” phenomenon in the layout of medical resources, which indirectly affected patients' evaluation and preference for PMIs (43, 44). A reason is the different strength of policy implementation in the two provinces. Qinghai Province strictly controls the referral rate; for example, it is stipulated that the referral rate of general township health centers does not exceed 60%. Residents must be first visited in PMIs and hold the “Qinghai Province Employees and Urban and Rural Residents Medical Insurance Tiered Treatment Referral Approval Form” before they can be referred to higher level hospitals, otherwise medical expenses will not be reimbursed (45). Beijing did not explicitly control the referral rate of PMIs, while only the elderly and residents of working age were first visited in PMIs, and the referral process is more flexible (46). Another reason is the different policy of family doctor contract services. Beijing adopts flexible contracting service period of 1, 2, and 3 years, and the contracting service fee is no <100 yuan per year. Qinghai Province is contracted once a year and the contracting service fee is 70 yuan per year.

There was no statistically significant differences for patients' willingness of first visit in PMIs in Qinghai and Fujian. According to our research, two main reasons were found. First, Fujian and Qinghai have a higher number of PMIs per capita. Fujian has 6.25 PMIs per 10,000 people, and Qinghai has 9.94 PMIs per 10,000 people. Second, Fujian's family doctor contracting service is an important initiative to realize community-based first visit. For example, Xiamen City has introduced the “three doctors co-manage patients” (general practitioner, health manager and specialist) family doctor service model. The primary healthcare reform in Xiamen has led patients to visit the PMIs more frequently (22, 47). In Qinghai, economic constraints and accessibility of PMIs have prompted patients to choose PMIs for their first visit (48). The per capita income of residents in Qinghai Province is low. When residents experience illnesses, they may prefer to seek treatment in less costly PMIs. So these reasons lead to high and similar willingness of patients in Fujian and Qinghai provinces to make their first visit in PMIs.

Our study found that recognition of the community first visit policy was associated with patients' willingness of first visit in PMIs. This is consistent with findings of previous study that the higher patients' recognition of the community first visit policy, the greater possibility of patients' willingness to make their first visit in PMIs (49). The results showed that only 11.7% patients recognized the community first visit policy (“completely recognize” and “partly recognize” were regarded as recognize), and the majority of patients were not. This finding was much lower than previous studies conducted in Shenzhen (72.03%), Wuhan (43.06%), Nanjing (59.5%), China (37, 50, 51). Compared with previous studies, there are two major reasons for this difference: First, there is a correlation between understanding of the policy and recognition of the policy for patients. Patients who are more understanding of the policy are more willing to recognize it. Second, sample variability leads to different results. This study's sample was drawn from tertiary and secondary hospitals and community health centers, whereas the samples of previous studies were all drawn from community health centers. Wenya Yu et al. found that patients who supported the community first visit policy were more willing to refer downward to community health centers (52). This study and Wenya Yu's study combined indicate that patients' recognition of the community first visit policy indirectly impacted the utilization of primary health care services. Although there was no statistical difference between understanding of the community first visit policy and patients' willingness of first visit in PMIs, only 9.9% patients know to the community first visit policy. Hence, besides improving the construction of the hierarchical medical treatment system, it is necessary to strengthen the promotion and interpretation of relevant policies for residents.

The influence of the medical insurance differential reimbursement policy was not associated with patients' willingness of first visit in PMIs. However, the medical insurance differential reimbursement policy is one of the main reasons why patients were willing to first visit PMIs. There are three possible reasons for this situation: First, patient' s recognition and trust of service capability of PMIs is an important driver for patients to first visit PMIs. Under the premise that service capacity of PMIs can meet patients' medical needs, and the government implements the medical insurance differential reimbursement policy, patients will be willing to first visit PMIs. However, the current service capacity of PMIs in China is uneven and cannot meet the growing needs of patients (8). When the gap in service quality between PMIs and large hospitals is obvious, the medical insurance differential reimbursement policy cannot effectively guide patients to make their first visits in PMIs. Hence, the precondition for moderating effect of the differential reimbursement policy is the homogenization of quality of medical services. Second, medical services are a rigid demand, and patients' demand for medical services is less affected by price. In order to maximize health benefits, patients will choose better medical care. Third, the influence of the medical insurance differential reimbursement policy is influenced by many factors, such as family financial status, severity of illness, and education (53). Thus, the influence of the medical insurance differential reimbursement policy in this study was not associated with patients' willingness of first visit in PMIs, probably because of the influence of other variables in the model.

Binary logistic regression results showed that patients who were very satisfied with medical technology of PMIs were more willing to make their first visit in PMIs than those who were generally satisfied. However, few patients (19.7%) were satisfied with the medical technology of PMIs. Previous studies have shown that the medical technology of PMIs plays a decisive role for patients' willingness to make first visits (31, 54). This study also found that the major reason for patients' unwillingness of first visit in PMIs was distrust of the medical skills of PMIs. Patients may compare the medical technology of PMIs with that of large hospitals. However, compared with large hospitals, PMIs do not have advantages in medical equipment, medical personnel, and medical environment. Another reason may be that the medical service capacity of PMIs cannot meet patients' medical needs. The Chinese government proposed the Medical Alliance Policy in 2017 (5), which aims to promote the downward transfer of focus and sinking of resources in health care and enhance the capacity of primary care services through establishing partnerships among PMIs, secondary, and tertiary hospitals. Secondary, tertiary hospitals supervise and guide medical work of primary health care institutions, and regularly provide targeted training and exercise for primary care staff. However, it seems that implementation of the Medical Alliance Policy has not achieved the expected effects, and patients' preference for primary care providers has not significantly changed. Since launching the health care reform plan in 2009, the Chinese government has formulated several policies, and increased funding for primary care, from 19 billion yuan in 2008 to 197 billion yuan in 2018 (55). A series of plans to strengthen the construction of the primary health care team, to alleviate the residents “difficult and expensive medical treatment” played an important role.

### Strengths and Limitations

This study is a multi-province investigation study with a large sample, which increases the generalizability in the Chinese setting. One of this article's outstanding strengths is understanding the impact of policies on patients' willingness of first visit in PMIs, and provided preliminary evidence to enhance the utilization of primary health care. Meanwhile, there are several limitations in this study. First, only partial factors were included in this study, but there are many factors associated with patients' willingness of first visit in PMIs (severity of illness, doctor-patient relationship) (56, 57). Second, this study only included the community first visit policy and health insurance reimbursement policy, but not the family doctor contracting policy. Third, all data were obtained from questionnaires, which might cause response bias. Fourth, this study used convenience sampling to select patients to administer questionnaires, so selection bias was unavoidable.

## Conclusions

Patients' willingness of first visit in PMIs is associated with different provinces, recognition of the community first visit policy, and satisfaction with the medical technology of PMIs. As influenced by factors such as local economic conditions, distribution of health resources, and differences in policy formulation, there are certain regional differences in patients' willingness of first visit in PMIs. This study explored the factors associated with patients' willingness of first visit in PMIs, and provided preliminary evidence to enhance the utilization of primary health care. To increase patient' rate of visits in PMIs, it is important to improve service capacity and quality of PMIs and change residents' attitudes for PMIs. Meanwhile, it is recommended that different provinces have to develop appropriate relevant health policies based on the actual situation. Using easy-to-understand language to publicize the advantages of relevant health policies in the community, strengthening residents' understanding and recognition of relevant health policies and guiding them to seek medical treatment in PMIs are important. The aim of all these measures is to achieve community-based first visit via guidance, considering that mandatory community-based first visit in China cannot be achieved overnight. In the background of the Health China strategy, the Chinese government is exploring health management-oriented primary health service policies by bundling medical treatment, medicine and health insurance through implementing outpatient capitation payment. Therefore, in the future, it is necessary to explore whether capitation payments for outpatient can promote patient visits to PMIs. This is also the essential way to promote community-based first visit, two-way referral and hierarchical treatment.

## Data Availability Statement

The original contributions presented in the study are included in the article/Supplementary Material, further inquiries can be directed to the corresponding author/s.

## Ethics Statement

The studies involving human participants were reviewed and approved by the Ethics Committee of Capital Medical University. The patients/participants provided their written informed consent to participate in this study (NO.Z2020SY117).

## Author Contributions

JL and JY contributed to conception and design of the study, and manuscript revision. NZ, HY, and HZ collected data and organized the database. JL wrote the first draft of the manuscript. NZ and HZ performed the statistical analysis. All authors contributed to reading the manuscript and approved the submitted version.

## Funding

This study was supported by the National Natural Science Foundation of China (grant number: 71603175).

## Conflict of Interest

The authors declare that the research was conducted in the absence of any commercial or financial relationships that could be construed as a potential conflict of interest.

## Publisher's Note

All claims expressed in this article are solely those of the authors and do not necessarily represent those of their affiliated organizations, or those of the publisher, the editors and the reviewers. Any product that may be evaluated in this article, or claim that may be made by its manufacturer, is not guaranteed or endorsed by the publisher.
