# Supplementary material for: Patients' Willingness of First Visit in Primary Medical Institutions and Policy Implications: A National Cross-Sectional Survey in China
Source: Front Public Health. 2022 Apr 1;10:842950. doi: 10.3389/fpubh.2022.842950 (PMC9010779; doi:10.3389/fpubh.2022.842950)
Supplement: Supplementary file 1 [file Data_Sheet_1.PDF]

Supplementary material

A questionnaire on patients' willingness to make their first visit in primary medical institutions

Part 1 Basic Information

1. What is your gender?

①Male ②Female

2. What is your age?(years)

①<45 ②45-54 ③55-64 ④≥65

3. What is your level of education?

①Junior or below ②Senior high school ③Bachelor or above

4. Where is your registered permanent residence?

①The city’s downtown ②The city’s suburbs ③Out-of-town

5. How long have you residence in the city?(years)

①<1 ②1-2 ③≥2

6. Are you an outpatient or an inpatient?

①Outpatients ②Inpatients

7. What is your current annual income?(yuan)

①<80000 ②80000-150000 ③>150000

8. What are your average actual monthly medical expenses?(yuan)

①≤300 ②301-800 ③>800

9. Do you have a chronic disease?

①Yes ②No

Part 2 Attitude toward primary medical institutions

10. How satisfied are you with the medical technology of community health centers/stations?

①Not satisfied ②Less satisfied ③Generally

④More satisfied ⑤Very satisfied

11. How satisfied are you with the service attitude of community health centers/stations?

①Not satisfied ②Less satisfied ③Generally

④More satisfied ⑤Very satisfied

12. Have you experienced a visit to primary medical institutions in the past year?

①Yes ②No

13. What are the main reasons why you were willing to visit community health centers/stations?(Choose the three options you think are most important)

①Closer to home ②Higher medical insurance reimbursement rate ③Treatment environment fit for recovery ④Want to be referred to a large hospital via a PMIs ⑤Large hospitals cooperate closely with PMIs ⑥Short waiting time ⑦More detailed consultation ⑧Access to family doctor services ⑨Access to the long prescription policy ⑩Other

14. What are the main reasons why you were unwilling to visit community health centers/stations?(Choose the three options you think are most important)

①The referral process wastes time ②Distrust of the medical skills of PMIs ③Fewer checkup items ④Fewer medical items reimbursed ⑤Inadequate variety of drugs ⑥Cannot be hospitalized in PMIs ⑦Poor medical environment ⑧Other

15. Are you willing to make your first visit in the community and then be referred to a higher-level hospital via the community health center/station?

①Very unwilling ②More unwilling ③Indifferent

④More willing ⑤Very willing

Part3 Level of understanding of relevant policies

16. Do you understand the community first visit policy?

①Yes ②No

17. How much do you recognize the community first visit policy?

①Not recognize ②Mildly recognize ③Moderate

④Partly recognize ⑤Completely recognize

18. Do you understand of the medical insurance differential reimbursement policy?

①Yes ②No

19. Do you think the medical insurance differential reimbursement policy directs patients to visit community health centers/stations?

①No impact ②Less impact ③Moderate

④More impact ⑤Greatest impact
